# An update around the evidence base for the lower extremity ultrasound regional block technique

**DOI:** 10.12688/f1000research.7199.1

**Published:** 2016-01-26

**Authors:** Andrea Fanelli, Daniela Ghisi, Rita Maria Melotti

**Affiliations:** 1 Department of Medical and Surgical Sciences, University of Bologna, Bologna, Italy; 2 Department of Anaesthesia and Postoperative Intensive Care, Istituto Ortopedico Rizzoli, Bologna, Italy

**Keywords:** Ultrasound, lower extremity blocks, lumbar plexus, sacral plexus

## Abstract

Ultrasound guidance currently represents the gold standard for regional anesthesia. In particular for lower extremity blocks, despite the heterogeneity and the lack of large randomized controlled trials, current literature shows a modest improvement in block onset and quality compared with other localization techniques. This review aims to present the most recent findings on the application of ultrasound guidance for each single lower extremity approach.

## Introduction

The use of ultrasound applied to peripheral nerve blocks has been the object of several randomized controlled trials (RCTs) and reviews in the last two decades ^1– 3^. Despite the heterogeneity of RCTs comparing ultrasound guidance to other localization techniques in terms of type of block, anesthetic agents, and control groups, a recent systematic review published by Liu has shown a moderate superiority of ultrasound guidance for the majority of the evaluated block characteristics ^2^. The effectiveness of ultrasound guidance showed by Liu was previously pointed out by Lewis *et al.* ^4^. The authors conducted a meta-analysis showing that ultrasound guidance produces a superior success rate in terms of readiness for surgery after sensory and motor test and fewer blocks requiring analgesic supplementation or conversion to general anesthesia compared to other localization techniques ^4^. Unfortunately, only six of the 32 studies considered by Lewis *et al.* were related to lower extremity blocks ^4^.

The interest and the evidence related to the use of ultrasound guidance applied to lower extremity blocks have been growing in the last few years ^2^. In fact, Liu ^2^ identified nine new RCTs evaluating lower extremity blocks, beside the eight pre-existing studies included in the first review in 2010 ^5^. Based on the analyzed data, the author shows that ultrasound guidance provides a modest improvement in block onset and quality when applied to lower extremity blocks ^2^. In particular, three studies reported a faster onset by 5 to 14 minutes if ultrasound was used to perform lower extremity blocks ^2^.

This review aims to present the most recent findings on the application of ultrasound guidance for main lower extremity blocks and approaches.

## Anatomy of interest for lower extremity peripheral nerve blocks

Ultrasound provides the possibility to directly visualize the nerves, vessels, needle, and local anesthetic distribution in the majority of patients ^6^, but it does not replace the full comprehension of the anatomy that still represents the foundation for safe and successful blocks and for the right management of the anesthetic/analgesic plan.

The lumbar plexus is an anastomotic complex formed by the anterior branches from L1 to L4 roots ^7^. The lumbosacral trunk is formed by the anterior branch of L5 together with an anastomotic branch from L4. The lumbar plexus has a triangular form: its base is represented by the lumbar vertebrae and its apex is formed by the union of the third roots with the ascendant rami of the fourth ^7^. The lumbar plexus lies anterior to the transverse process within the psoas muscle. All of the branches of the lumbar plexus emerge from the psoas muscle and leave the pelvis. The lumbar plexus gives origin to ﬁbers for the main trunks of the lower extremity: the femoral and obturator nerves, as well as for the sciatic nerve through the lumbosacral trunk ^7^.

The obturator nerve is formed by the L2−L4 roots. It leaves the psoas muscle at the level of the sacroiliac joint. In the pelvis, it runs close to the ureter and the internal iliac artery with an outward and downward direction. The obturator nerve leaves the pelvis and enters the thigh at the upper part of the obturator foramen, reaching the obturator groove, where in the majority of cases it divides into an anterior and a posterior branch ^7^. The anterior branch runs in front of the adductor brevis and adductor magnus muscles and behind the adductor longus. The posterior branch runs between the adductor brevis and adductor magnus muscles ^7^.

The femoral nerve is the main terminal branch of the lumbar plexus and is formed by the roots of L2 to L4 ^7^. After its origin, the femoral nerve runs in the iliopsoas groove and enters the thigh toward the inguinal ligament, where it generally divides into its terminal branches ^7^. In the femoral triangle, the nerve is localized laterally to the artery, deep to the fascia lata and to the fascia iliaca, and on the anterior aspect of the iliopsoas muscle ^7^. The saphenous nerve is a terminal branch of the femoral and it is of clinical interest for knee and leg surgeries ^8^. At the femoral triangle, the saphenous nerve is located within the posterior plane, and it innervates the skin of the medial part of the knee and the anteromedial part of the leg and foot. The saphenous nerve is also involved in the innervation of the knee joint ^7^.

The sacral plexus is formed by the anterior sacral roots from S1 to S3 and by the lumbosacral trunk ^7^. The sacral plexus transverses the sciatic foramen lying anterior to the piriformis muscle. At this level, it is separated from the visceral structures of the pelvis by the pelvic aponeurosis. In the parasacral region, the sacral plexus provides two branches involved in the innervation of the hip joint, potentially relevant for the anesthesia and analgesia of patients undergoing complex hip surgeries ^7^. In the thigh, the sciatic nerve runs between the semitendinosus and semimembranosus muscles medially and the biceps femoris muscle laterally ^7^. From the middle of the thigh to the popliteal crease, the common peroneal and tibial nerves give off branches to the posterior part of the knee joint. Inside the popliteal fossa, the tibial and common peroneal nerves provide a medial and a lateral sural cutaneous nerve respectively ^7^.

In terms of nerve anatomy, the ratio of connective and neural tissue in the sciatic nerve increases from proximal to distal, to the point of creating a thick paraneural sheath around the nerve ^2, 9^. This sheath plays an important functional role in the spread and clinical effect of the local anesthetic injected during a sciatic block.

## Lumbar plexus block

The application of ultrasound guidance for the lumbar plexus block is still challenging due to the depth of the nerve structures and the presence of the ‘acoustic shadow’ of the transverse processes ^10, 11^. In clinical practice, ultrasound guidance is commonly used for the lumbar plexus block in combination with nerve stimulation, and it can be applied either for pre-procedural scanning or as real-time guidance. Sonoanatomical studies have shown that it is possible to visualize the transverse processes, vertebral body, psoas major muscle, erector spinae, quadratus lumborum, lower pole of the kidney, peritoneum, aorta, and vena cava ^12, 13^. As previously described, the continuous psoas compartment block can be performed under real-time ultrasound guidance using either a longitudinal or a transversal scan and an in-plane needle approach ^1^. When a longitudinal scan is preferred, a curved array transducer is used to perform the first subcostal scan to localize the inferior kidney pole. The second scan is dedicated to visualizing the sacral promontory in long axis as a sonoanatomical landmark, which allows one to ascend cranially to L2–L3, counting the shadows of the transverse processes ^14^. A reliable catheterization of the lumbar plexus can be achieved after identifying the transverse processes of L2–L3 and the psoas muscle lying between them ^1^. These sonoanatomical landmarks are always reproducible, whereas visualization of the lumbar nerve roots might be challenging with both transverse and longitudinal approaches. The advantage of the technique is that the needle is inserted in-plane, on a sagittal plane, to avoid any medial direction and potential epidural localization of the catheter, even if repeated needle contact with transverse processes could represent a drawback of the technique. The aid of a nerve stimulator can help in improving the reliability of the described approach. Recently, Karmakar *et al*. published a prospective case series evaluating the feasibility of a modified transverse scan of the lumbar paravertebral region with the ultrasound beam being insonated through the lumbar intertransverse space and directed medially toward the intervertebral foramen ^15^. The target vertebral level for the lumbar plexus block is identified by locating the lumbosacral junction on a paramedian sagittal scan and then counting cranially to locate the lamina and transverse processes of the L3, L4, and L5 vertebrae. The modified approach introduced by Karmakar *et al*. is then performed with the transducer positioned 4 cm lateral to the midline in the transverse orientation at the L3−L4 intervertebral level ^15^. The aim is to identify the lumbar nerve root close to the intervertebral foramen. The needle is inserted in-plane at a point 4 cm lateral to the midline and medial to the transducer and then it is slowly advanced under ultrasound guidance to the posterior aspect of the psoas muscle until either needle–lumbar plexus contact is visualized or an ipsilateral quadriceps muscle contraction is elicited ^15^.

## Suprasacral parallel shift block

As reported by Bendtsen *et al*., anesthesia and analgesia for hip surgeries can be obtained with a single injection performed under ultrasound guidance reaching the terminal nerves of the lumbar plexus and the lumbosacral trunk; this new approach was denominated by the authors the suprasacral parallel shift block ^16^. The insertion site of the needle is in correspondence of the interspace between the upper rim of the sacral ala and the lower border of the transverse process of L5. The local anesthetic solution has to be injected into the compartment behind the psoas muscle where the lumbosacral trunk, the obturator, and the femoral nerve are located. To perform the suprasacral parallel shift block, a curved array probe (2−5 MHz) is required and has to be initially placed parallel to the iliac crest and then moved medially until the sacral bone is recognized. Afterwards, the probe has to be rotated until the upper margin of the sacral bone, the transverse process of L5, and the interspace between the two bony structures come into view. The needle is inserted with an out-of-plane approach perpendicular to the skin and advanced until it penetrates the lumbosacral ligament and loss-of-resistance is perceived ^16^.

The ultrasound-guided suprasacral parallel shift block was recently tested in terms of effectiveness for anesthesia of the terminal nerves of the lumbar plexus compared with a classic lumbar plexus block ^17^. Moreover, the effectiveness of the two approaches to obtain anesthesia of the lumbosacral trunk was evaluated. This RCT conducted on volunteers showed that the suprasacral parallel shift block is at least as effective as the lumbar plexus block for blockade of the terminal nerves of the lumbar plexus and is significantly more effective for blockade of the lumbosacral trunk ^17^. Unfortunately, the suprasacral parallel shift block is not superior to the lumbar plexus technique to provide anesthesia of all dermatomes from L2 to S1 ^17^.

## Fascia iliaca compartment block

The fascia iliaca compartment block, first described by Dalens *et al*. ^18^, has been reported to effectively block the three major terminal nerves of the lumbar plexus: the femoral, the obturator, and the lateral cutaneous nerves. The fascia iliaca block has been the object of attention in the last few years due to its potential role as first-line pain therapy for patients presenting to the emergency department with a proximal femoral fracture ^19^. The use of ultrasound to perform the fascia iliaca block was found to be superior when compared with the traditional approach using ‘loss-of-resistance’ to identify the correct plane, but still requires high volumes of local anesthetic ^20^. At the level of a theoretical line drawn between the pubic tubercle and the anterior superior iliac spine, a linear high-frequency probe is used to identify the fascia iliaca and to guide the needle to the correct plane in transverse, short-axis view ^20^.

## Femoral nerve block

The femoral nerve block represents the gold standard for analgesia of patients undergoing major knee surgeries ^21^. With a high-frequency linear probe, the femoral nerve appears in a transverse section, as a triangular hyperechoic region, which lies lateral to the artery, deep to the fascia lata and to the fascia iliaca, and on the anterior aspect of the iliopsoas muscle ^22^. Different authors have been evaluating whether the local anesthetic distribution and/or the catheter tip position may reduce the incidence of motor block maintaining the analgesic effect ^23, 24^. Ilfeld *et al*. showed that an anterior catheter tip positioning may increase cutaneous sensory block versus a posterior catheter tip placement, without increasing motor block ^23^. Moreover, Szűcs *et al*. showed that depositing the local anesthetic only anteriorly to the nerve results in fewer needle redirections and greater patient satisfaction compared to surrounding the neural target circumferentially ^24^. Farag *et al*. compared ultrasound guidance alone with either ultrasound guidance plus needle stimulation or ultrasound guidance plus catheter stimulation, in terms of postoperative pain, for insertion of femoral nerve catheters in more than 400 patients undergoing total knee arthroplasty ^25^. The authors showed how ultrasound guidance alone represents the best approach to femoral perineural catheters ^25^.

## Saphenous nerve block

In the last few years, the adductor canal block, a variant of the saphenous nerve block, has been presented as an alternative to the femoral nerve block in patients undergoing total knee replacement. In fact, the saphenous nerve block preserves quadriceps function in contrast to the femoral nerve block due to the fact that it is a pure sensory nerve ^26, 27^. A recent RCT has shown how the ultrasound-guided block of the saphenous nerve at the level of the adductor canal is superior to the block at the distal trans-sartorial level in terms of success rate, faster onset time, and better nerve visibility under ultrasound ^28^. Nevertheless, it is important to recognize that the nerve to the vastus medialis also lies in the proximal portion of the adductor canal, possibly resulting in undesired motor weakness when a more proximal approach to the saphenous nerve is preferred. A cadaver study determined that in the majority of subjects (72.5%), the most distal point where it’s possible to see the nerve to the vastus medialis is where it transfixes the muscle proximal to the site where the saphenous nerve passes over the anterior surface of the superficial femoral artery to arrange medially to the vessel ^29^. This anatomical knowledge is extremely important for deciding where to perform the block of the saphenous nerve, whether proximally or more distally.

## Obturator nerve block

Several approaches have been described to block the obturator nerve. The ultrasound-guided technique can be performed in-plane or out-of-plane and the nerve can be blocked before or after its bifurcation into the anterior and posterior branches. The patient is placed in a supine position and their thigh is externally rotated. The probe commonly used is a high-frequency linear probe, which is located perpendicular to the skin in an opposite position to angle between the inguinal crease and the adductor longus. This approach gives the possibility of correctly visualizing the structures of interest: the pectineus muscle, the adductor longus muscle, the adductor brevis muscle, and part of the adductor magnus muscle ^30, 31^. In this way, the operator can identify the anterior and the posterior divisions of the obturator nerve: the first one is located between the adductor longus and brevis muscles, while the second one is below the adductor brevis muscle ^30, 31^. Sinha *et al*. showed that an ultrasound-guided block in which 50% of the local anesthetic solution is injected between the pectineus muscle and the adductor brevis muscle and the rest of the anesthetic solution between the adductor brevis and the adductor magnus muscle allows one to obtain a mean reduction of muscular strength of 82% in almost all the patients evaluated (93%) ^32^. Comparable results were achieved by Manassero *et al*. They conducted a RCT to determine whether interfascial spread of local anesthetic can supplant nerve stimulation as the end point for local anesthetic injection during ultrasound-guided obturator nerve block after the division of the obturator nerve ^33^. The end point of their in-plane injection was correct interfascial spread of local anesthetic, defined as spread within the muscle interface, resulting in separation of target muscles. The authors demonstrated that this ultrasound-guided intrafascial injection is comparable with nerve stimulation ^33^. 

## Parasacral sciatic nerve block

In the case of complex hip surgery, the gold standard is represented by the parasacral approach to the sciatic nerve due to the fact that with this proximal approach it is possible to block both the superior gluteal nerve and the nerve to the quadratus femoris muscle. Nevertheless, in comparison with more distal approaches to the sciatic nerve, the parasacral one is characterized by a potential increased risk of damage of the internal iliac vessels, ureter, rectum, and superior gluteal artery if the needle is advanced beyond the nerve ^2^. Typically, the patient is placed in Sims’ position and a low-frequency curved probe is applied. In Taha’s approach, the small axis of the sciatic nerve is located at the posterior border of the ischium that usually can be identified as a curved hyperechoic line at this level ^34^. The nerve lies deep to the piriformis muscle, lateral to the inferior gluteal vessels ^34^. Whereas there is a high variability in the quality of image among patients, the nerve stimulator is considered useful to increase the reproducibility in this deep block.

## Trangluteal sciatic nerve block

The transgluteal approach is easier and safer compared with the parasacral one. At the transgluteal level, ultrasounds allow one to easily identify the greater trochanter, the ischial tuberosity, and the sciatic nerve, which is located between them ^35^. At this level, if the physician inserts the needle beyond the nerve, he/she would commonly hit the bone and the only real risk is represented by the puncture of the inferior gluteal artery vessels. The patient assumes the Sims’ position and the operator uses a curved array low-frequency probe. The sciatic nerve is found beneath the gluteus maximus muscle halfway between the greater trochanter laterally and the ischial tuberosity medially ^35^.

## Subgluteal sciatic nerve block

As previously reported, at the thigh level, the sciatic nerve can be recognized through the paraneural sheath that surrounds it. Two recent studies have analyzed the functional aspect of this space ^36, 37^. The first study, conducted on cadavers, showed that after injection of dye under the paraneurium, there was both distal and proximal spread along the nerve ^36^. Instead, if the dye was injected outside the sheath, the spread was more much limited ^36^. Ultrasound guidance allows an optimal image of the dissection ^36^. The second study compared the injection of local anesthetic outside or under the paraneurium in patients undergoing subgluteal sciatic nerve block ^37^. A three-dimensional reconstruction was made after the accomplishment of the block to quantify the spread of the local anesthetic solution along the nerve. Joining this technique with a clinical correlation about the quality of sensory block, it was possible for the authors to prove that an injection under the paraneurium allows better diffusion of local anesthetic and higher block efficacy than outside the paraneurium layer ^37^. Karmakar *et al*. ^38^ have definitively confirmed Andersen’s results ^36^ and stated that the target for local anesthetic injection is the subparaneural space. To perform subgluteal or popliteal sciatic nerve blocks a linear high-frequency probe is usually sufficient. Although the patient can be in the ventral or supine position, the prone position is preferred. The ultrasound nerve localization can guide the choice of the puncture point in the posterior thigh. The local anesthetic should be placed all around the nerve between the epineurium and paraneurium. As reported by Krediet *et al*., the discrimination of the position of the needle tip is not always easy ^39^. It is possible to identify the intraneural or extraneural needle tip position using an injection test of 0.5 mL, but even experts missed one of six intraneural injections ^39^. A subparaneural injection not only accelerates the onset time but also increases the duration of the sensory blockade compared with circumferential extraneural injection ^40^. If an extra-paraneurium injection is performed, the circumferential injection of local anesthetic around the nerve provides a higher success rate and shorter onset time than local anesthetic deposition at a single location next to the nerve ^41^. On the contrary, when approaching the nerve at a subparaneural level, single injection or triple injections result in comparable success rates and total anesthesia related times ^41^.

## Ankle block

Ultrasound-guided ankle block is frequently used and effective for foot surgeries. Compared with the conventional approach, the application of ultrasound increased the success rate and reduced the onset time of the ankle block ^42– 44^. To identify the four nerves of sciatic origin, a linear high-frequency probe is used. The tibial nerve is usually located between the medial malleolus and the Achilles tendon posterior to the posterior tibial artery and veins ^45^. On the anterolateral aspect of the ankle next to the anterior tibial artery, the deep peroneal nerve can be identified. The superficial peroneal nerve is scanned before it becomes subcutaneous 10 to 15 cm proximal to the lateral malleolus. The sural nerve is located between the lateral malleolus and the Achilles tendon, close to the saphenous vein if it is visualizable ^45^.

## Conclusion

In conclusion, in the last few years, ultrasound guidance has allowed us to use different approaches targeted to the clinical context, optimizing differential block. A better comprehension of the sonoanatomy has suggested new perspectives in local anesthetic distribution around the target nerves, especially for the sciatic nerve, which is the most studied at the level of the popliteal fossa.

Despite the heterogeneity of the studies analyzing the clinical impact of the ultrasound applied to the lower extremity blocks, current evidence shows a moderate improvement in performance time, patient procedural comfort, and in some cases even onset times compared to other localization techniques.
